# Food Delivery Platform: A Potential Tool for Monitoring the Food Environment and Mitigating Overweight/Obesity in China

**DOI:** 10.3389/fnut.2021.703090

**Published:** 2021-07-29

**Authors:** Na Cong, Ai Zhao, Peng Gong

**Affiliations:** ^1^Ministry of Education Key Laboratory for Earth System Modeling, Department of Earth System Science, Tsinghua University, Beijing, China; ^2^Vanke School of Public Health, Tsinghua University, Beijing, China; ^3^Education Ecological Field Station for East Asian Migratory Birds, Tsinghua University, Beijing, China; ^4^Departments of Geography and Earth Sciences, University of Hong Kong, Hong Kong, China

**Keywords:** food delivery platform, food environment monitoring, overweight/obesity, health intervention, China

## Introduction

A report on the status of nutrition and chronic diseases among Chinese citizens, released on November 23, 2020, revealed that 50.7% of Chinese adults are overweight and obese (1). The rate is considerably higher than that in 2002 (29.9%) and 2012 (42%) (2). Overweight and obesity decrease life expectancy and increase health expenditure (3). According to estimates from the Organization for Economic Co-operation and Development (OECD), there is likely to be about 3 years reduced life expectancy among the OECD, EU28, and G20 countries, and 92 million people are predicted to die prematurely due to overweight between now and 2050 (3). In addition, 311 billion dollars will be spent per year (according to the USD Purchasing power parity) treating overweight and related conditions in OECD countries (3). Nonetheless, in 2014, the number of obese people in China rose to first place in the world, and the number of severely obese people has increased to second place globally (4). Overweight and obesity have overtaken undernutrition as the primary burden despite a huge double concern of malnutrition in China (2, 5).

The intricate determinants for overweight and obesity are discussed in many studies (6–13). Biologically, overweight and obesity are caused by an imbalance between energy intake and energy expenditure (14). Many case studies discussed the associations between food consumption and obesity in adults in China (15–32). Recent research shows that rapid behavioral shift to consumption of processed food with low nutritional value and high energy (33, 34) and widespread decreased physical activities (6, 35–38) contribute to the epidemic of overweight and obesity. Dietary patterns represent a broad picture of food consumption (39). The dietary pattern in China was relatively healthy in 2010 compared with that of other countries (40). However, China has since then quickly transformed toward unhealthy diets (40) under the increasing adoption of western dietary patterns (41). Such shifts are also related to changes in the food environment (11).

**Figure 1:**
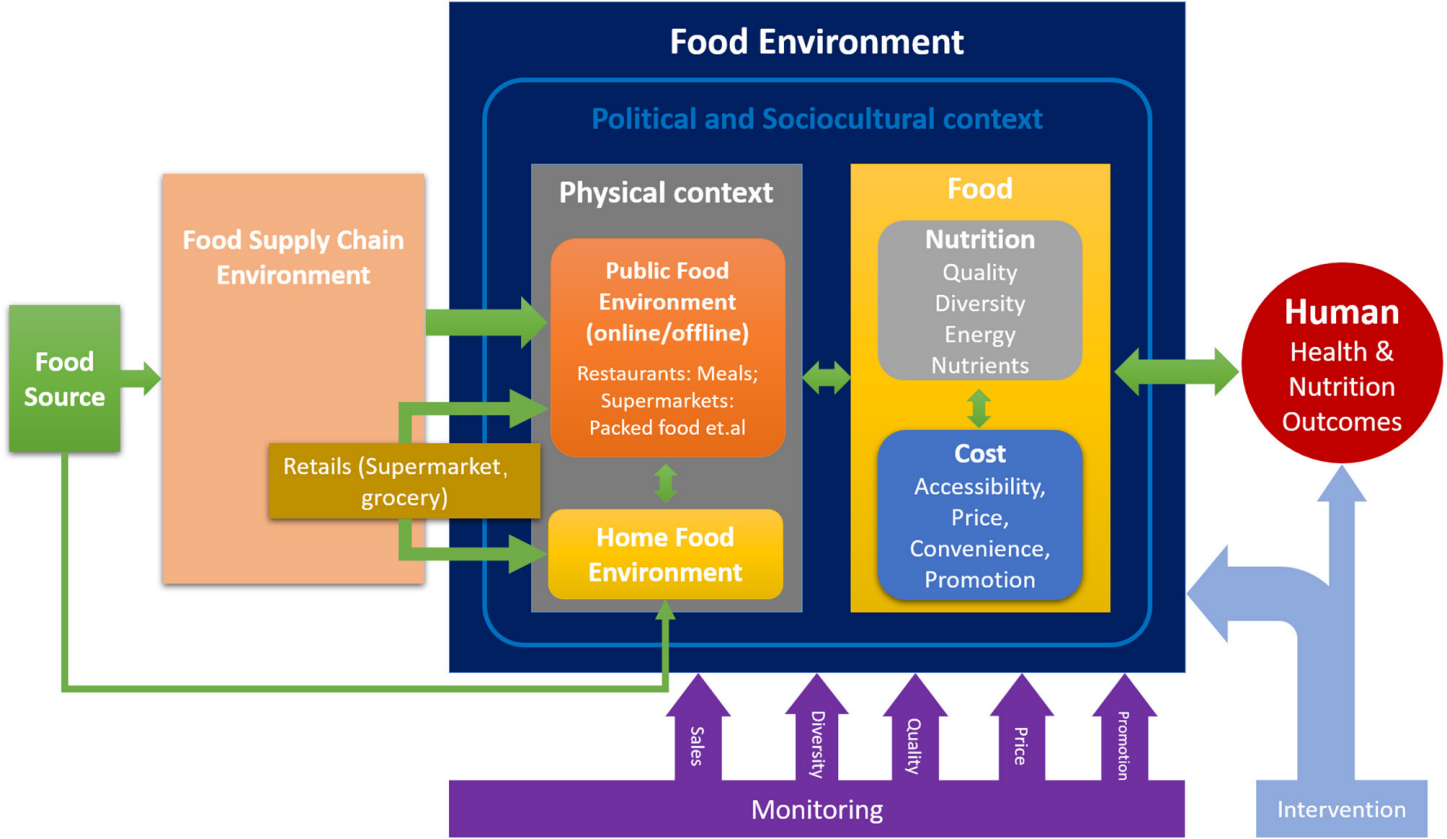
A conceptual framework of the food environment.

Food environment, as shown in Figure 1, is defined as a group of physical, economic, policy, and sociocultural surroundings, opportunities, and conditions that impact the food and beverage choice of individuals (42–44). It plays a crucial role in promoting healthy and sustainable eating as an interface among humans and broader food systems (45, 46). Research on the food environment has become a priority in addressing the double burden of malnutrition (47). However, the complexity of the food environment, diverse dietary behavior, and the absence of representative Body Mass Index (BMI) data, relevant behavioral and environmental monitoring data (48, 49), and all-around lack of food environment research in low- and middle-income countries (LMIC) (46) make it difficult to obtain a comprehensive understanding of the environmental drivers of obesity to achieve a consistent conclusion. Thus, building a monitoring system can facilitate research on obesity and its connections to the food environment to mitigate its rate. In the following, we would like to unfold this discussion from two aspects: the changes in the food environment in China and the potential for a food delivery service platform (FDSP) to monitor the food environment and intervene in the obesity issue in China.

## Changes in Food Environment in China

Cereals and vegetables with little animal meat are considered the traditional Chinese diets (5). Before the reform and opening-up of China, due to the food shortages, people could only obtain food using food coupons. Low intake of cereal (50), animal meat, and edible oil and fat were the main dietary pattern. After a market economy developed in China, it was correspondingly easier to access food as food production became liberalized. Initially, the consumption of cereals increased rapidly but has declined slowly since 1978. The consumption of meat rose nearly four times between 1979 and 1992 compared to 1952–1979 (50). After joining the World Trade Organization (WTO), the rise in economic development, urbanization, opening markets to western countries, and technological advances have led to a rapid transition in lifestyle among Chinese citizens (51). Particularly, over the last 10 years, the wide application of living service software (the software integrating all kinds of living service outlets online to satisfy the needs of customers) and an efficient delivery system, have enabled Chinese people with improved access to most household goods (e.g., clothes, packaged food, fruit or other raw food from its origin, and almost anything one needs) at the national/local scale and to ready-to-eat food at the local/neighborhood scale. The distance between consumers and grocery stores or restaurants has virtually degenerated to zero under the impact of express delivery services, even though some food outlets are thousands of kilometers away.

For the home food environment, rural people grew and produced food by themselves or exchanged food with their neighbors before 2000, while most urban citizens purchased food from local markets or from mobile vendors who are farmers. Currently, under the impact of e-commerce and the promotion of poverty alleviation policies, a new e-commercial model, the “Farm to Table” strategy, could deliver fresh food harvested from farms directly to consumers. The COVID-19 induced explosion of fresh food consumption has occurred on e-commercial platforms. The number of users of fresh food e-commercial applications had risen to 257 million by June 2020 (52). Food cooked at home comes not only from local markets but also from national and even international markets. Due to population mobility, urbanization (53), and the booming of e-commerce, dietary habits for families across the country are being influenced by a multilevel food environment.

For food environments away from home, the convenience and time-saving of takeaway food have led to a considerable expansion in demand (51). By the end of 2019, the number of users ordering takeaway food from applications was 458 million, accounting for almost one-third of the total population of China (54). This number covers an age range from 18–40 years and is similar to that of other countries (55). Moreover, the average annual orders had risen dramatically, from 21.4 orders per person per year in 2017 to 32.5 orders per person per year in 2019. During the lockdown imposed by COVID-19, more than half of the population had ordered food from an FDSP at least once during a 24 h period dietary survey (56). This transition shows that the personal dietary habits had shifted from a home food environment to a modern public food environment (as shown in Figure 1), which provides more cooked food, prepared food materials, or fresh food from outside through online and offline public business services. The public food environment is a type of “food environment away from home,” which involves more online social environments. Although it is too early to tell whether or not this transition is healthy, it is an excellent opportunity to tackle the overweight and obesity problems caused by the change in the food environment. The internet technology involving many customers and producers is also a helpful tool to investigate the linkage between food delivery service and obesity risk, a subject not well studied (11).

## Potential of FDSP to Monitor the Food Environment and to Intervene in the Obesity Pandemic in China

Obesity is not only an issue for the individual but also one of the most pressing social challenges (57). Though increasingly more official reports advocate for deeper and broader research, there is still no national research on the spatial distribution of overweight and obese populations at sufficiently high resolution. There is also a lack of analysis of social determinant factors like food environment at the national scale. It is, therefore, necessary to build a system that could capture more changes in the food environment to support investigations on the associations/causality among, behaviors and health status, intervention strategies related to population health, particularly overweight and obese problems. Of the e-commercial platforms, FDSPs are the most suitable to monitor and intervene in diet habits for the following reasons:

### Broader Spatial Coverage

Regarding spatial coverage, the food delivery service covers most cities of various administrative levels in China, and there are no differences in restaurant density across cities in China (54). The national monitoring could help researchers seek out key factors impacting human diet by comparing dietary patterns and food environment in different areas and make relevant policies on the food environment at a low cost. This is a great supplement to the traditional dietary survey (5).

### Long-Term Food Consumption and Food Environment Monitoring

On one hand, the FDSP continuously records the food ordered for every meal. The researcher can analyze the individual dietary preference and at what point in time their diet behaviors change based on the long-term monitoring data. On the other hand, the FDSP collects attributes of online food outlets and the dynamic change in the food environment such as price, opening time, and promotion. The long-term monitoring from the FDSP could assist researchers to conduct time series analysis on the association between diet behavior and food environment to determine the best timing for an individual diet intervention.

### Fewer Confounders for Measuring the Food Environment

The online food environment is easier to monitor compared to restaurants and home environments. The restaurant food environment is complicated. The attitude of waiters/waitresses, the design of the dining room on light, decoration, tableware, all kinds of promotions, and food styles of restaurants would impact food consumption as the primary potential factor. These multiple factors could confound each other in some ways. However, the online food environment can focus on the nutrition of food, the price, and the preferred tastes of the individual. This conforms to the requirements of the research and helps make simple intervention strategies.

### Accurate and Personalized Intervention on the Human Diet

E-commerce companies invest large amounts of effort to develop algorithms to reconstruct the characteristics of users, accurately capture personal preferences, and match consumer demand. The work that the e-commerce companies have done is helpful to detect whose diet is unhealthy. According to user characteristics and diet preference, an accurate and personalized intervention strategy could be developed under the guidance of the Dietary Guidelines for Chinese Residents.

Despite the above advantages, the FDSP is a new commercial model which has only been developed in recent years, so there are some limitations in applying this kind of platform to assist in understanding the food environment and obesity research. First, food ingredients and their nutritional attributes are unavailable *via* these online systems. The detailed food components in a meal could not be obtained automatically since not all food outlets currently provide such information. Only some food outlets are prepared to share the name and weight of food to attract more consumers. Second, the FDSP only records food consumption by meal and cannot capture the full spectrum of food intake of a person. Thus, it is difficult to depict an individual dietary pattern because of discontinuous food consumption.

Some of the above limitations could be solved by technologies or policies in the future. However, the potential for applying data from FDSPs in support of the “Health First” national strategy is far from being fully exploited. How to predict the diet-related health risk of users based on the characteristics, preferences, and consumption records of users, the best time to carry out the intervention, and the most suitable method to prevent this kind of risk are vital questions to be answered in the context of the food delivery platform. Unfortunately, in China, few studies have considered these research questions. The FDSP has already begun intervening in the human diet (58) by changing the online food environment. For example, the FDSP helps some food outlets improve their viewability to guide customers to these food outlets by designing some rules. The risk of being exposed to high-fat, high-sugar, and other unhealthy food may increase if these rules are not developed according to the dietary guidelines. However, we know little about how these rules are developed by the FDSP firms, and the effects and potential health and social consequences of these interventions, especially with regards to obesity issues (11). Under the pressure of comprehensive chronic disease prevention, the urgency to begin this line of research based on FDSPs is consequently of vital importance.

## Conclusions

Until now, most countries in the world have not been able to prevent the global trend of rising obesity (4, 49). Although cities are key to controlling health challenges successfully, more detailed strategies and specific actions are needed to attain the desired results since the “Healthy China 2030” plan only provides broad guidelines (59). As the first line of defense of a healthy city, the food environment will be the main target of diet-related actions or strategies. With the development of the Local Living Service platform, more modern lifestyles are shaped by major e-commercial platforms such as Taobao, Meituan, Pinduoduo, and Jingdong in China. Most of the aspects in Living Services related to human health are recorded, analyzed, and applied to achieving more significant commercial benefits. Although some of these platforms have started to promote the integration with health under the guidance of the “Healthy China 2030” policy, the scientific research based on data recorded on these platforms is not sufficient to form effective intervention strategies to promote human health. Hence, faced with the largest group of overweight and obese people in the world, the Chinese government should make more effort to build the most advanced defense system and long-term monitoring network, in cooperation with private big data companies, to detect changes in nutrition and chronic disease burden (60) to achieve the goal of “Healthy China 2030.” Now is the best time to act.

## Author Contributions

PG provided the original idea. NC wrote the first draft of the manuscript. AZ contributed to the discussion of the idea and revised the paper. All authors contributed to the article and approved the submitted version.

## Conflict of Interest

The authors declare that the research was conducted in the absence of any commercial or financial relationships that could be construed as a potential conflict of interest.

## Publisher's Note

All claims expressed in this article are solely those of the authors and do not necessarily represent those of their affiliated organizations, or those of the publisher, the editors and the reviewers. Any product that may be evaluated in this article, or claim that may be made by its manufacturer, is not guaranteed or endorsed by the publisher.
